# Spontaneous pnemomedastinum in acute severe asthma

**DOI:** 10.4103/0972-5229.68224

**Published:** 2010

**Authors:** N. M. Aleemuddin, Farah Bahmed

**Affiliations:** **From:** Department of Respiratory Medicine, Deccan College of Medical Sciences, Kanchan Bagh, Santoshnagar, Hyderabad, India

**Keywords:** Spontaneous pneumomedastinum, cervical medastinotomy, tracheostomy, 100% oxygen, Hammans sign

## Abstract

Spontaneous medastinal emphysema, as a complication of acute severe asthma, is an uncommon entity. It usually runs a benign course and resolves spontaneously without any surgical intervention. Recognition of this complication is critical, as it has to be differentiated from other life threatening ones including oesophageal rupture, Boerhave’s syndrome, acute coronary syndrome and pulmonary embolism. This case is being presented to emphasize its recognition in the differential diagnosis of complications arising from acute severe asthma and to present its management strategy in detail.

## Introduction

Substernal chest pain during an asthma attack, subcutaneous emphysema in the neck and over the chest and the Hamman’s sign on auscultation should raise the suspicion of a spontaneous pneumomedastinum.[1–4] Air lucency in the soft tissues of the neck, along the cardiac silhouette on CXRPA view and in front of the heart on lateral view confirms the diagnosis.[5–6] This condition usually gets resolved spontaneously and requires only close observation and management of the asthma. 100% oxygen, cervical medastinotomy and tracheotomy are required only in cases of cardiopulmonary distress. Spontaneous pneumomediastinum is defined as the presence of free air in the mediastinal structures without an apparent precipitating cause. Its occurrence ratio is approximately one case per 10,000 hospital admissions,[7] and thus, it may not be very familiar to most of the physicians. Asthma alone accounts for 22-50% spontaneous pneumomedastinum cases, as mentioned in various studies.[8–9] There are several studies published recently about pneumomedastinum in acute severe asthma.[10–13] Asthma is very commonly encountered in clinical practice but medastinal emphysema as its complication is an infrequent problem. Review of literature reveals that less than 100 cases have been published till now. We report a case of spontaneous pneumomedastinum complicating acute severe asthma in an adult male.

## Case Report

**Figure 1:**
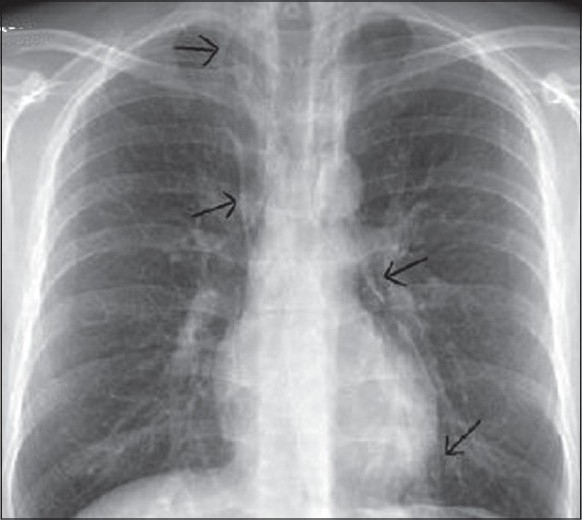
Thin lucency of air seen along borders of heart indicated by arrows

**Figure 2:**
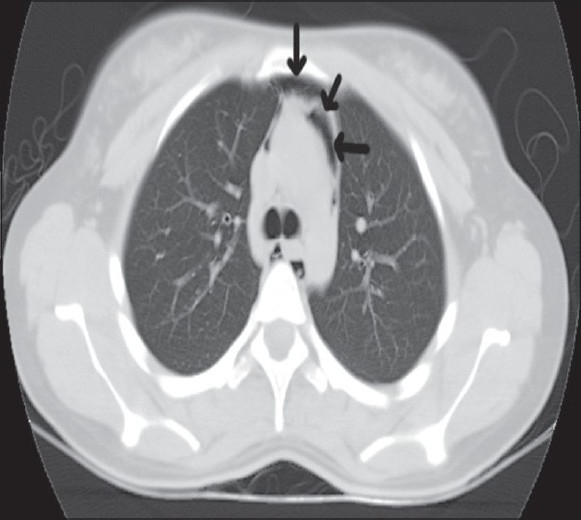
Rounded lucencies seen within medastinal pleura surrounding the medastinal structures indicated by arrows

**Figure 3:**
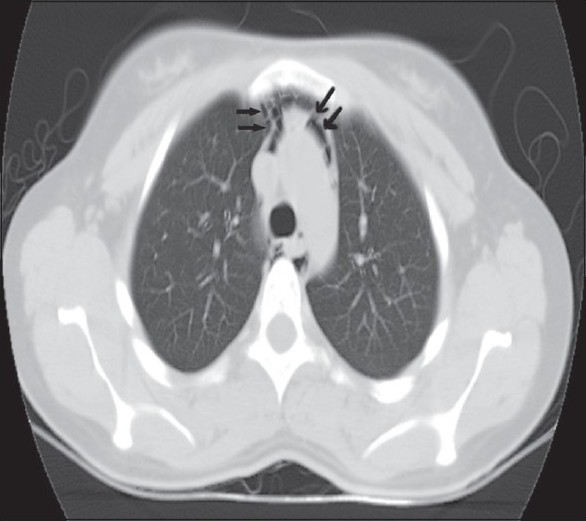
Rounded lucencies seen within medastinal pleura surrounding the medastinal structures indicated by arrows

**Figure 4:**
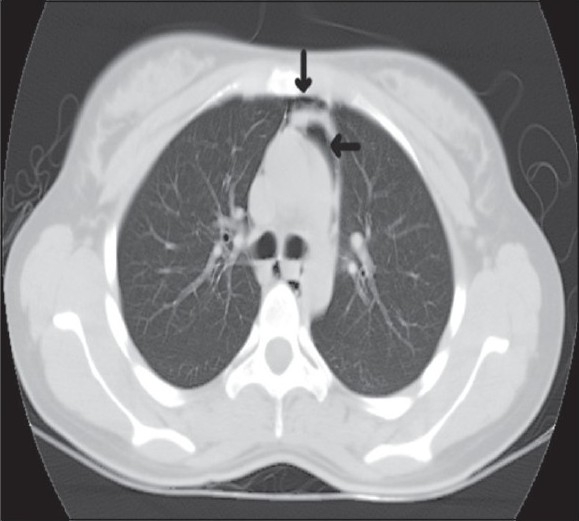
Rounded lucencies seen within medastinal pleura surrounding the medastinal structures indicated by arrows

A 37-year-old known asthmatic male patient presented with sudden onset of retrosternal chest pain of two hours duration. He was asymptomatic two days back, and then developed productive cough, breathlessness and wheezes, which was not associated with fever or chest pain. Two hours prior to presentation, he developed sudden onset of chest pain while driving, which was not associated with trauma, vomiting or sweating. Chest pain was severe, pleuritic, nonradiating and piercing type with no relieving or aggravating factors. There was no associated dysphagia suggesting any oesophageal perforation. Physical examination revealed subcutaneous emphysema over the neck and the anterior chest wall. He was febrile with his blood pressure being 120/80 mm Hg, pulse 100/min, RR 18/minute regular and ECG within normal limits. Auscultation revealed bilateral expiratory wheeze with prolonged expiration and no adventitious heart sounds were heard. CXR [Figure 1] revealed pneumomedastinum without pnemothorax or obvious rib fractures. CT scan chest [Figures 2–4] revealed pneumomedastinum without any other abnormality. He was managed with oxygen, nebulisation, hydrocortisone, oral bronchodilators and pain medication. Patient improved symptomatically and the chest pain disappeared after 48 hours. CXR and CT scan after seven days showed no abnormality.

## Discussion

The case described above highlights an unusual complication of acute severe asthma. In asthma, spontaneous pneumomedastinum occurs due to the existence of a decreasing pressure gradient between the alveoli and the lung interstitium that can result in alveolar rupture. This pressure gradient can be produced by decreasing pleural pressure, which occurs when the Mueller’s maneuver is performed in bronchial asthma or by decreasing interstitial pressure which occurs in intense work of breathing and vasoconstriction or by increasing intraalveolar pressure. In acute severe asthma, explosive rise of intra alveolar pressure arising from partial or complete obstruction of airways causes rupture of the alveoli and seeping of air into the interstitium centripetally (Macklin effect).[14] Air travels along the perivascular sheaths and further dissects into the medastinum producing medastinal emphysema. Cardiopulmonary distress may complicate the situation if air compresses large veins in the medastinum. No intervention is necessary in most cases; severe cases with hemodynamic or respiratory instability could require intervention.[2–5] In case of cardiopulmonary distress, treatment options include 100% oxygen to increase the diffusion pressure of nitrogen in subcutaneous tissues that results in an increased rate of reabsorption, thereby relieving pressure symptoms. In life threatening situations cervical mediastinotomy can be performed by making incisions in the supraclavicular fosse posterior to the sternocleidomastoid muscles and in the suprasternal notch. Furthermore tracheotomy can be performed, that not only decompresses the medastinum but also decreases the intra alveolar pressure thereby preventing further seeping of air into medastinum.
